# Dramatic enhancement of superconductivity in single-crystalline nanowire arrays of Sn

**DOI:** 10.1038/srep32963

**Published:** 2016-09-06

**Authors:** Ying Zhang, Chi Ho Wong, Junying Shen, Sin Ting Sze, Bing Zhang, Haijing Zhang, Yan Dong, Hui Xu, Zifeng Yan, Yingying Li, Xijun Hu, Rolf Lortz

**Affiliations:** 1 State Key Laboratory for Heavy Oil Processing, PetroChina Key Laboratory of Catalysis, China University of Petroleum, Qingdao 266580, China; 2 Department of Physics, Hong Kong University of Science and Technology, Clear Water Bay, Kowloon, Hong Kong; 3 Institute of Physics and Technology, Ural Federal University, Russia; 4 Department of Chemical and Biomolecular Engineering, Hong Kong University of Science and Technology, Clear Water Bay, Kowloon, Hong Kong

## Abstract

Sn is a classical superconductor on the border between type I and type II with critical temperature of 3.7 K. We show that its critical parameters can be dramatically increased if it is brought in the form of loosely bound bundles of thin nanowires. The specific heat displays a pronounced double phase transition at 3.7 K and 5.5 K, which we attribute to the inner ‘bulk’ contribution of the nanowires and to the surface contribution, respectively. The latter is visible only because of the large volume fraction of the surface layer in relation to the bulk volume. The upper transition coincides with the onset of the resistive transition, while zero resistance is gradually approached below the lower transition. In contrast to the low critical field *H*_c_ = 0.03 T of Sn in its bulk form, a magnetic field of more than 3 T is required to fully restore the normal state.

Nano structuring materials can change their physical properties dramatically. Nanotechnology thus provides the opportunity to tailor new materials with improved or entirely new characteristics. In the case of superconductors, the confinement of Cooper pairs in the nanometer geometry is in most cases unfavorable for applications: for example, it may cause a suppression of the critical temperature *T*_c_^1^,^2^, or a finite resistance at finite temperatures^3^,^4^,^5^,^6^,^7^. Nevertheless, some rare cases where the constrained geometry of nanoscale superconductors can create interesting effects have been reported. For example, a slight increase in the superconducting transition temperature was observed in nanoparticles of In^8^,^9^, Tl^8^, and Ga^10^,^11^,^12^ and Pb nanobelts^13^,^14^. Furthermore, some enhancement of the critical fields^9^,^11^,^13^,^14^,^15^,^16^ and unusual magnetoresistance oscillations have been observed^13^,^14^. In addition, we recently reported a large improvement of the critical field and onset critical temperature in arrays of parallel 5 nm thin Pb nanowires^17^. A similar *T*_c_ increase was observed more recently in Pb microspheres^18^, while previous work on Pb nanowires reported rather a suppression of superconductivity below a certain critical thickness^19^,^20^,^21^.

The density of states at the Fermi level is one of the crucial parameters that determines the critical temperature in a classical superconductor. The exploration of finite size effects to enhance critical temperature and the superconducting energy gap value by tuning the Fermi level to a region where the density of states is high has been studied in detail both theoretically^22^,^23^,^24^,^25^,^26^,^27^ and experimentally^28^,^29^,^30^,^31^,^32^, especially for the case of nano-grains of superconducting materials with some coupling^29^, e.g. through the Josephson effect. It has been demonstrated that both the size of the nano-grains and the intra-grain coupling strength represent crucial parameters for the superconducting characteristics^22^. However, in reduced dimensionality, thermally induced fluctuations of the superconducting order parameter are greatly enhanced^33^,^34^. They lower the critical temperature dramatically below which zero resistance is found, although phase incoherent Cooper pairs may exist at much higher temperatures^29^. In a two-dimensional superconductor, the superconducting phase transition occurs in form of a Berezinskii-Kosterlitz-Thouless transition, which occurs below the bulk critical temperature^35^,^36^,^37^. Quasi-one-dimensional (quasi-1D) superconductivity is found in nanowires which are thinner than their superconducting coherence length^38^,^39^. In 1D superconductors, according to the Mermin-Wagner theorem, thermally induced order parameter phase slips will cause finite resistance at any *T* > 0 K^33^,^34^. The resistance then shows only a continuous decrease below the temperature where the Cooper pairs form^3^,^4^,^5^,^6^,^7^,^39^,^40^. The dimensional crossover from a 3D superconductor in thick nanowires to a 1D fluctuating superconducting state as a function of the nanowire thickness has been studied in detail in single crystalline Sn nanowires and nanowire arrays^39^,^40^. It has been shown theoretically^41^,^42^,^43^,^44^,^45^,^46^,^47^,^48^ and experimentally^17^,^49^,^50^ that a long-range-ordered state with zero resistance may be formed when many 1D superconducting nanowires are arranged in close proximity to form a regular array. A transverse Josephson or proximity coupling can then suppress the phase slip processes and mediates zero resistance at finite temperatures^51^.

In this paper, we report a dramatic increase of the onset critical temperature when elemental Sn is brought into the form of networks of randomly oriented weakly coupled freestanding nanowires, while a true zero-resistance state is preserved below 2 K.

## Results

**Figure 1:**
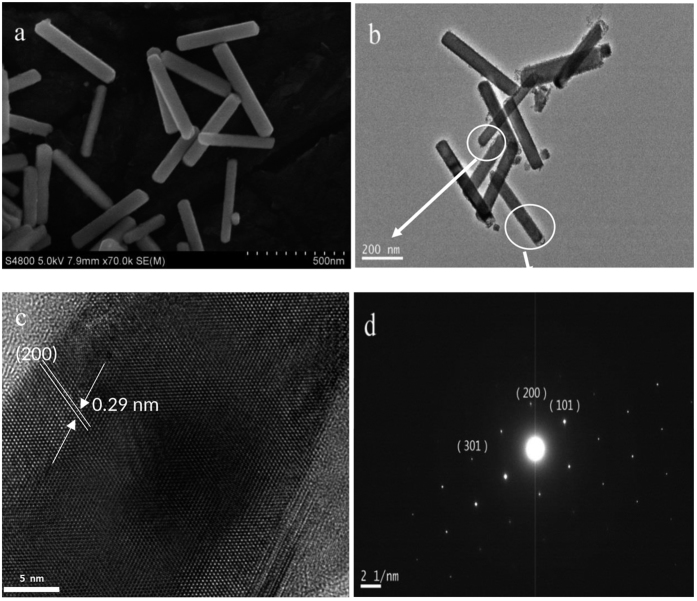
(**a**) SEM image of Sn nanowires, (**b**) TEM image of Sn nanowire and its (**c**) HRTEM image and (**d**) ED pattern.

The growth process and characterization of the Sn nanowires is described in detail in the Experimental Section at the end of this article. In Fig. 1 we represent TEM and SEM images, showing the Sn nanometer of typical thickness of 60–70 nm in diameter and with average length of 500 nm, as shown in Fig. 1a,b. Prior to the experiments, the nanowires were suspended in an ethanol solution. For the experiments, a macroscopic amount of a few hundreds of micrograms was dried. The material forms then a powder consisting of small micrometer size grains containing the networks of loosely bound bundles of nanowires. The lattice-resolved HRTEM image (Fig. 1c) reveal a lattice spacing of 0.29 nm corresponding to the (200) planes of β-Sn. The HRTEM images and the ED pattern (Fig. 1d) unambiguously demonstrate the single-crystal structure with [100] orientation. Further characterization data demonstrating the high degree of crystallinity at different positions of a nanowire can be found in the Supplementary Materials.

### DC Magnetization

**Figure 2:**
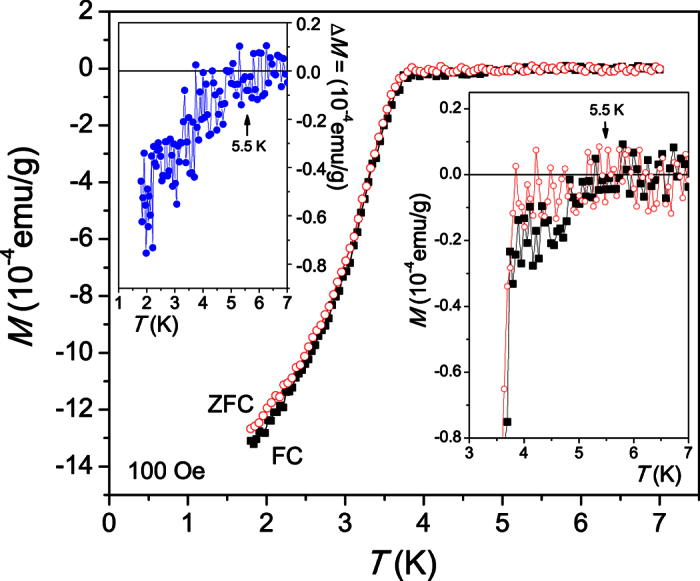
DC magnetization of a macroscopic sample containing the Sn nanowires measured under ZFC (squares) and FC (circles) conditions. The right inset shows an enlargement of the transition onset, which shows that a weak Meissner signal develops below ~5.5 K. The left inset shows the difference Δ*M* = *M*_*ZFC*_ − *M*_*FC*_ between the ZFC and FC branches.

Figure 2 shows the DC magnetization measured on a macroscopic sample of total mass of 843 μg under zero-field cooled (ZFC) and field cooled (FC) conditions in an applied field of 100 Oe. A clear Meissner effect develops below 3.8 K. The transition is very continuous, which is likely a consequence of the quasi-1D nature of the nanowires^17^. Some irreversibility is visible between the ZFC and FC branches. The right inset show an enlargement of the transition onset, which shows that a very weak diamagnetic signal develops already below ~5.5 K, which is 1.8 K above the bulk critical temperature of elemental Sn. In the left inset the difference Δ*M* = *M*_*ZFC*_ − *M*_*FC*_ between the ZFC and FC branches is shown, which shows that traces of irreversibility persist up to at least 5 K.

### Specific heat

**Figure 3:**
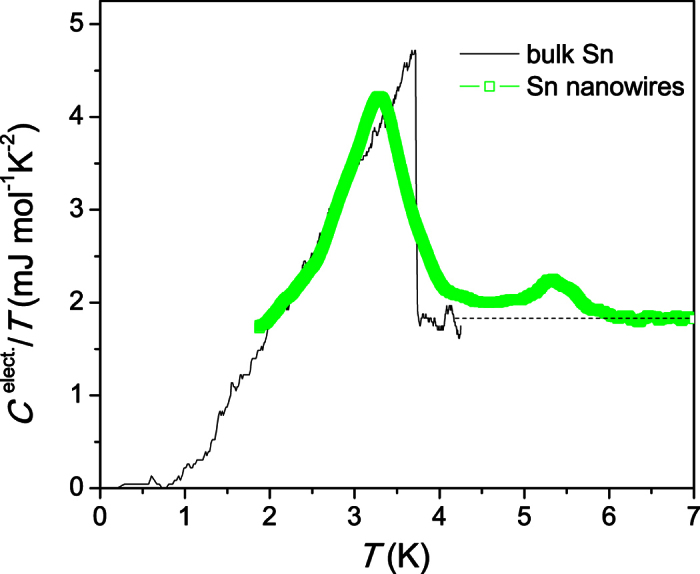
The electronic heat capacity *C*^electr^/*T* at the superconducting transition of Sn nanowires (open squares) as a function of temperature in zero field. The additional data (line) with the sharp jump at 3.7 K represents literature data of bulk Sn shown for comparison^54^.

The specific heat of a macroscopic sample of total mass of 100 μg containing the loosely bound Sn nanowire network was obtained with our dedicated modulated temperature AC micro-calorimetric technique^52^, as described in more detail in the Experimental Section. The electronic contribution was obtained in a standard way^53^ with help of a measurement of the normal state data in a magnetic field of 14 T, which is sufficient to suppress superconductivity. Figure 3 shows *C*^electr^/*T* of the Sn nanowires in comparison to literature data^54^ for bulk Sn. The broad superconducting anomaly in the specific heat of the Sn nanowires differs significantly from the sharp jump-like anomaly of bulk Sn. The center of the main transition at 3.7 K coincides roughly with the jump observed in bulk Sn. However, the transition anomaly is much more continuous and shows a more symmetrical shape (albeit with a rather sharp kink at the maximum). A fluctuation tail extends well above the *T*_c_ of bulk Sn up to at least 4.5 K until the second smaller transition anomaly occurs with a broad jump at 5.5 K. Below ~6 K, *C*_electr_/*T* is significantly larger than the normal state Sommerfeld constant, which together with the fact that the anomaly is suppressed concurrently with the main transition by an applied field indicates its superconducting origin. Both transitions are fully suppressed by the 14 T magnetic field. The presence of two distinct transition anomalies agrees perfectly with the magnetization data where a weak transition onset was observed at 5.5 K. The specific heat shows that this transition onset corresponds to a significant volume fraction of the sample.

**Figure 4:**
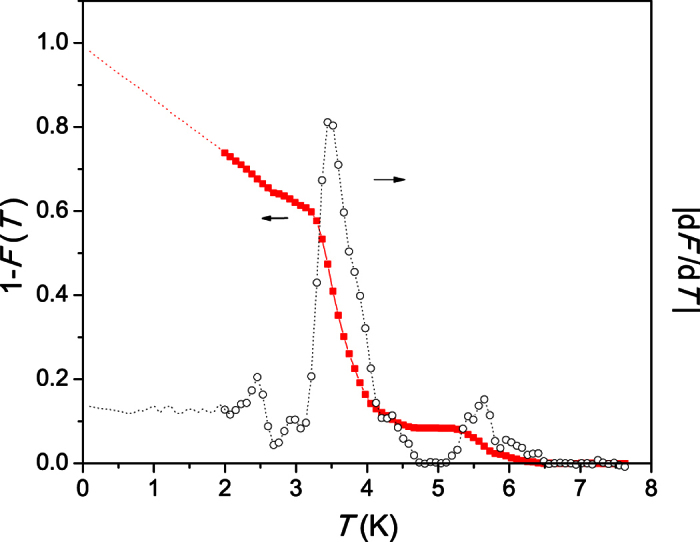
Superconducting volume fraction of a macroscopic sample of Sn nanowires 1 − *F*(*T*) and the corresponding *T*_c_ distribution d*F*/d*T*^55^.

Using the standard Two Fluid Model, we split the electronic heat capacity into the Sommerfeld and superconducting components and so can derive the *T*_c_ distribution^55^ of the Sn nanowires. The 1 − *F*(*T*) and d*F*/d*T* correspond to superconducting volume fraction and the *T*_c_ distribution, respectively (Fig. 4). This deconvolution of the specific heat further illustrates the large width of the superconducting transition, which extends from 1.5 K to almost 6.5 K, with peaks occurring in d*F*/d*T* at 3.7 K and 5.5 K.

### Electrical transport

**Figure 5:**
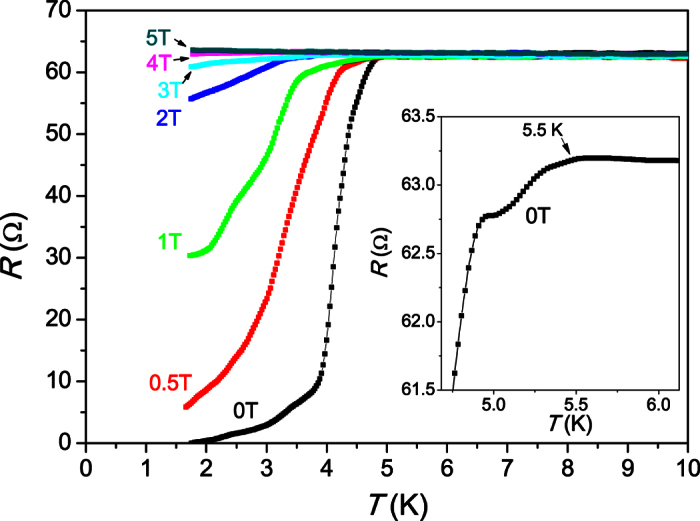
Electric resistance at the superconducting transition of a micrometer-size grain containing a network of many randomly aligned Sn nanowires in various magnetic fields. The inset shows an enlargement of the transition onset, which reveals that the onset of the superconducting transition is enhanced up to 5.5 K.

Figure 5 shows the electrical resistance of a network of a large number of randomly aligned nanowires in the form of a small grain of a few microns in size. We present the resistance instead of resistivity, since the precise current path in this device is unknown. In view of the 1D nature of the nanowires, the resistance drops surprisingly sharp with midpoint around 4.1 K. Below the main transition, the resistance remains continuously dropping until zero resistance is reached at ~2 K. Zooming to the onset of the transition (inset of Fig. 5) reveals that the resistance begins to drop significantly at 5.5 K, which is 1.8 K above the transition of bulk Sn. This agrees well with the previous results obtained from the heat capacity and magnetization and confirms the superconducting origin of the second transition anomaly at 5.5 K. Applying a magnetic field of 3 T is required to fully suppress the resistive superconducting transition, which contrasts enormously to the low critical field of 0.033 T of bulk Sn. Thus, the resistivity data demonstrates that nano-structuring of Sn provides an enormous enhancement of both, the onset *T*_c_ and the upper critical field.

**Figure 6:**
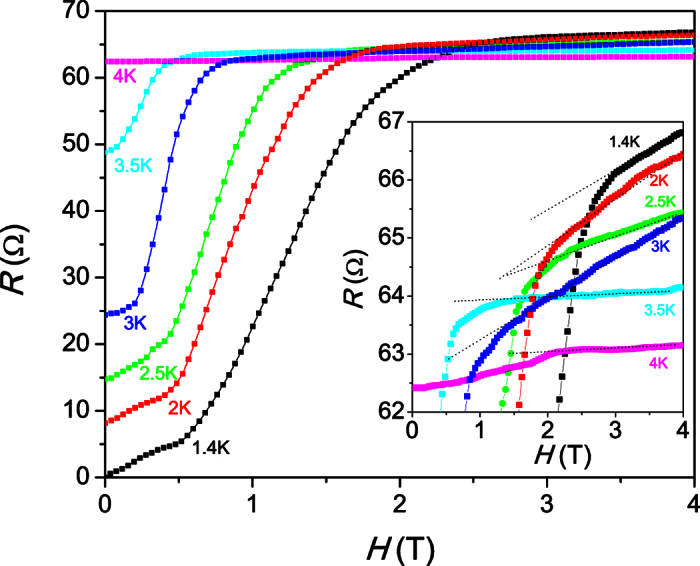
Magnetoresistance at various fixed temperatures. The inset shows an enlargement of the region of the onset of superconductivity at the upper critical field.

Figure 6 shows the magnetoresistance of Sn nanowires in field sweeps at different temperatures. At 1.4 K, the magnetoresistance increases starting from the lowest fields, slowly at first and more rapidly above 0.5 T, until the normal state resistance is approached at 2 T. We attribute this to the effect of strong phase fluctuations as a consequence of the reduced dimensionality of the nanowires: for nanowires in the 1D limit it is expected that zero resistance is only found in the limit of zero Meissner screening current density, while any finite current density causes phase slips in the superconducting order parameter^33^,^34^. At higher temperatures, the resistance starts from finite values and the critical field is gradually lowered until a flat curve is found above 4 K. This quite temperature-independent normal state resistance is likely the effect of the weak links between the nanowires.

Zooming in on the 4 K data (inset of Fig. 6) shows that traces of superconductivity persist at this temperature for at least up to a broad kink at ~2.2 T. This is in accordance with the weak signature of superconductivity in the zero field resistance data up to 5.5 K.

## Discussion

Superconducting nanowires have been studied in detail in the past two decades. In early work on granular metal nanowires the focus was mostly on a strong to weak localization transition^6^. More recent work on single-crystalline nanowires focused on the 3D to 1D dimensional crossover in single-crystalline nanowires and nanowire arrays^17^,^39^,^40^. Our experiments show a strong enhancement of the onset *T*_c_ and a high upper critical field of Sn, when it is brought in the form of thin nanowires. Both are known effects in low dimensional superconductors^8^,^9^,^10^,^11^,^12^,^13^,^14^, although the enhancements are rarely as strong as what we observe. We have reported a similar enhancement of superconductivity in ultrathin Pb nanowires previously. In the case of Sn, Herzog *et al*.^6^ have reported already back in 1996 resistance data of much thicker granular Sn nanowires, which showed *T*_c_ onsets somewhat above 4 K, although that work had a different focus and the effect may have been caused rather by the granularity, while our nanowires are single crystalline. Nevertheless, our macroscopic sample, which consists of a large number of loosely bound Sn nanowires, shares many similarities to granular superconductors, which have been reported to show similar enhancements of their superconducting properties^56^,^57^,^58^,^59^,^60^. *T*_c_ enhancements from 1.2 K up to 3 K have been observed for example in granular Al films^61^. Two major mechanisms have been proposed as explanation for their *T*_c_ enhancement. Thomson and Blatt^25^ have demonstrated that a quantization of electronic motion in very thin superconducting films can have an increasing effect on the transition temperature, and the same mechanism has been attributed to the *T*_c_ enhancement of small isolated grains of some superconducting materials^58^. For very small superconducting quasi-zero-dimensional Sn nanoparticles, a similar factor has been reported recently that has been referred to as ‘Shell Effect’^30^. The discretization of the energy levels in very small particles causes fluctuations in the spectral density near the Fermi energy, which can eventually enhance the superconducting gap by large factors up to 60%.

The importance of the coupling of superconducting nanostructures for their superconducting parameters was demonstrated e.g. in recent experimental^29^ and theoretical work^22^: It was shown that in superconductors composed of weakly coupled nanograins, *T*_c_ can be enhanced by an increasing decoupling of the grains. At even weaker coupling *T*_c_ goes over a maximum where the transition crosses over into a phase-driven transition with a phase incoherent pseudogap persisting at higher temperatures^29^. In our nanowire network the nanowires are randomly aligned, which certainly causes a large distribution of different coupling strengths. This may further explain the large width of the superconducting transition observed in the specific heat.

A second mechanism was proposed to be an enhanced effective electron-phonon interaction near the surface of a superconductor^62^. Our samples are of similar dimensions in the lateral direction. The effect of quantization of electron motion would unlikely cause a double superconducting transition as observed in the specific heat of our Sn nanowire samples. On the other hand, the presence of a thin surface layer with an enhanced *T*_c_ would perfectly fit as explanation. This additional transition in the specific heat and the onset of the resistive transition at 5.5 K are thus likely not related to a bulk *T*_c_, but rather attributed to the surface layer of the nanowires^17^. In such thin nanowires, the surface layer occupies a substantial volume fraction of the nanowires. The heat capacity is most sensitive to the Cooper pairing and the transition at 5.5 K represents a significant fraction of 10% of the total pairing signal (see Fig. 4). The pronounced double transition anomaly cannot be explained solely by enhanced superconducting fluctuations due to the nanoscale size of our Sn nanowires, but suggests that the two transitions arise from different regions within the nanowires. Our single-crystalline nanowires are uniform and have an average thickness of 60–70 nm. Therefore, one possibility to explain our data is that a surface layer having a thickness of up to 7 nm is influenced by the softening of the phonons near the surface. Of crucial importance here is that the nanowires are thin enough so that a significant portion of the volume is affected by the surface phonon softening. In addition, the curvature of the surface may represent another important ingredient for the *T*_c_ enhancement in these cylindrical nanowires^63^. Without these effects, the *T*_c_ of a superconductor would rather be expected to decrease with dimensionality^33^,^34^,^64^. We would like to point out that a superconducting surface is not at odds with the finite resistivity we observe in the regime between 3.7 K and 5.5 K: the surface represents a very thin hollow cylinder, somewhat like a rolled up 2D superconducting sheet. In such a small geometry no phase-coherent superconducting state with zero resistance can be formed. The resistance will be strongly governed by thermally activated phase slips in the order parameter. In addition, the strong fluctuations will entirely suppress the Josphson coupling between the wires, thus causing an additional strong interwire resistance.

The electrical resistivity of thin single-crystalline Sn nanowire of similar thickness and arranged in arrays has been studied previously by Tian *et al*.^39^,^40^, but no *T*_c_ enhancement was reported. One reason is certainly that the evidence from the electrical resistivity is very weak, and the detection of the high onset *T*_c_ requires strong zooming in onto the transition onset. Sufficient resolution to do this is provided by our AC measurement technique with an AC current source in combination with a digital lock-in amplifier (see ‘Methods’ section for details). Our strongest evidence comes from the heat capacity, which shows a pronounced double transition. From the resistivity alone the effect may be easily overseen, and is certainly not visible on the scale the data has been presented in refs 28 and 29. A further reason may be that Tian *et al*. used superconducting electrodes fabricated from bulk Sn. The proximity effect from the electrodes below their *T*_c_ may sharpen the observed resistive transition and make the tiny transition onset appear even less pronounced, while the anti-proximity effect^65^ above their *T*_c_ may suppress the surface superconductivity over the small length scale probed in the experiment. The contacts on our samples are established by non-superconducting metals and the resistivity has been probed on a macroscopic grain containing the nanowire network over a micrometer length scale. The anti-proximity effect is thus not expected to be able to suppress surface superconductivity over the entire current path probed.

**Figure 7:**
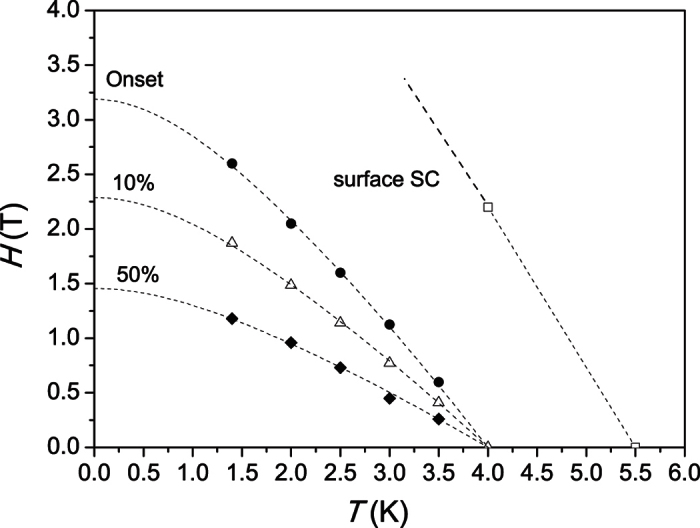
Magnetic field vs. temperature phase diagram as constructed from the magneto-resistance data in Fig. 6. Due to the broadness of the transition, three different criteria have been used to construct upper critical field lines: the ultimate high field transition onset, the field where the magneto-resistance dropped 10% from the normal state value and the transition midpoints (marked as 50%). The lines are fits according to the Werthamer-Helfand-Hohenberg (WHH) theory^66^. The additional line ending at 5.5 K is likely associated with surface superconductivity.

In Fig. 7, we attempt a rough estimate of the upper critical field at zero temperature with help of the jumps in the magnetoresistance data in Fig. 6. Note, that this value may be underestimated because of the sensitivity of electrical transport data on phase coherence, which likely gets gradually suppressed with increasing field. Due to the very large width of the transition, it is hard to provide a single value, and we use three different criteria to construct upper critical field curves: The ultimate onset of a superconducting signature in the high field limit where the data starts to deviate from the high field linear behavior, the field where the magnetoresistance dropped by 10% from the normal state value, and the midpoints of the broad jumps. The three resulting lines illustrate the continuous upper critical field crossover. The standard Werthamer-Helfand-Hohenberg (WHH) theory^66^ allows us to extrapolate the data to zero temperature and estimate the upper critical field *H*_c2_(0), which according to the different criteria fall in the field range between 1.4 T and 3.4 T. The 1.4 T value yields a reasonable upper estimate for a superconducting coherence length of 28 nm. The coherence length in our nanowires is thus certainly somewhat smaller than the average wire diameter, but still of the same order of magnitude needed for the observation of significant finite size effects. It is notable that the transition points of the two curves can be extrapolated to a *T*_c_(0 T) value of 4 K, which corresponds likely to the bulk *T*_c_ of the inner part of the nanowires.

To explain the large upper critical field in our nanowire arrays, several factors need to be considered. Bulk Sn is a classical type-I superconductor. A reduction of the electron mean free path in a superconductor decreases the superconducting coherence length *ξ* and can drive it into the type-II region with larger critical field *H*_c2_(0). This was shown, for example, for Pb doped with some Bi impurities^67^. The high critical field of our nanowires suggests a similar effect, caused by the confinement of the Cooper pairs in the quasi-1D structure. Our measurement techniques are not sensitive to probe whether the superconductor is of type-I, or type-II, but such high critical fields are usually found only in type-II superconductors, suggesting that the nano-structuring has indeed changed the characteristics of Sn from type-I to type-II. Moreover, a 1D material has an open Fermi surface without closed orbits. That suppresses the formation of vortices and thus the orbital limit for superconductivity^68^ at which the vortex cores overlap will be greatly enhanced. Eventually, the vortex cores only penetrate the nanowire network between the wires in form of Josephson vortices^69^, which have little effect on the Cooper pairs within the wires. A factor that then determines the upper critical field of such a low-dimensional superconductor may be the particularly strong spin orbital coupling^70^ in a metallic surface state band. In ultrathin Pb films with magnetic field applied parallel to the film it has been found that *H*_c2_(0) can be increased by two orders of magnitude^64^, and it has been demonstrated theoretically in the same paper that this is due to the strong spin-orbit scattering.

The fact that zero resistance is reached below 2 K shows that the Sn nanowires do not completely behave individually in our macroscopic powder sample, but probably show some coupling. The Josephson Effect may mediate this, if the nanowires are close to each other but without contact–or rather point contacts when they are in physical contact. The resistivity is thus likely most sensitive to this phase ordering effect, which establishes global phase coherence throughout the network of nanowires (probed by our experiment on the micrometer scale). Global phase coherence throughout the network is required for the resistance to drop to zero, while phase incoherent fluctuations that apparently form below 5.5 K, only cause the slight decrease in resistivity without a strong magnetoresistance effect in the high temperature regime. This coupling obviously then establishes a bulk phase-coherent state in the macroscopic sample, which may occur in a similar way as observed in Pb nanowires in SBA-15 matrices^11^ or in intrinsically quasi-1D superconductors such as Tl_2_Mo_6_Se_6_^49^ or Sc_3_CoC_4_^71^.

To conclude, we demonstrated that the superconducting characteristics of networks of freestanding Sn nanowires are substantially modified by nano-structuring, with a 1.8 K increase of the onset *T*_c_. Our nanomaterial thus represents an impressive example how superconducting parameters can be enhanced by employing finite size effects, while stabilizing phase fluctuations and preserving a zero resistance state through a weak coupling of the nano-sized building blocks in such an array of nanoparticles.

## Methods

Single crystalline Sn nanowires were synthesized using surfactant as soft template by a chemical reduction process. In a typical synthesis, 0.2 g sodium dodecyl sulfate (SDS) and 0.4 g tin sulfate were dissolved in 80 mL distilled water and heated to 303 K in a water bath under stirring. After 20 minutes, a sodium borohydride solution (0.02 g of NaBH_4_ in 5 mL of distilled water) was added. The nanowires formed after 20 minutes reaction. The resulting solution was separated by centrifugation and the precipitate was washed several times with distilled water and ethanol.

The morphology and microstructures of the resulting nanowires were characterized by a field emission transmission electron microscopy (TEM, JEM-2100UHR/200 kV), X-ray diffraction (XRD, Philips X’pert Pro Alpha 1 Diffractometer with Cu Kα radiation, λ = 1.5406 Å) and scanning electron microscopy (SEM, S-4800, Japan). The SEM and TEM images of the 60–70 nm in diameter thin nanowires of about ~500 nm in length is presented in Fig. 1. The lattice-resolved HRTEM image (Fig. 1c) clearly reveals the lattice spacing of 0.29 nm that corresponds to the (200) planes of β-Sn. The HRTEM images and the ED pattern (see Fig. 1d and additional data in the supplementary materials) unambiguously demonstrate the perfect single-crystalline structure with [100] orientation.

**Figure 8:**
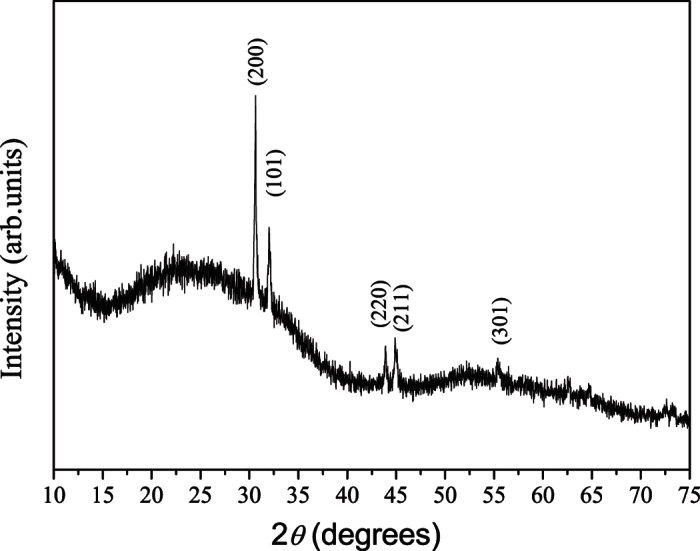
Higher-angle XRD data for the Sn nanowires.

The crystal structure of Sn nanowires was recorded by powder X-ray diffraction (XRD) as shown in Fig. 8. Diffraction peaks located at 30.6° and 32° can be attributed to the (200) and (101) planes of β-Sn (JCPDS No. 01-086-2265). It shows evidence of a preferred orientation with the (200) and (101) peaks. The intensity of the (200) peak for the as-made nanowires is much higher than that of the secondary (101) peak, which confirms the result from the TEM data that the growth direction of the Sn nanowires is highly preferred along the [100] direction.

Prior to the experiments, the Sn nanowires were suspended in ethanol. The nanowires were extracted from the solution and dried, which resulted in a powder consistent of small micrometer-sized grains of loosely bound and randomly oriented nanowire networks.

The DC magnetization of the Sn nanowire networks was measured with a commercial Quantum Design Vibrating Sample SQUID magnetometer in the temperature range from 1.8 to 10 K on a total mass of 843 μg powder under zero-field cooled (ZFC) and field cooled (FC) conditions in an applied magnetic field of 100 Oe.

The specific heat of the Sn nanowires was measured in a 14 T magnet cryostat with variable temperature inset (temperature range 1.4 to 300 K) with a standard modulated temperature AC micro-calorimetric technique^52^, which provides the required high relative resolution of Δ*C*/*C* ≈ 10^−5^ to resolve the tiny specific heat anomaly of these superconducting nanowires. A total mass of 100 μg sample in the form of powder was mixed with insulating GE 7031 varnish, which served as a thermal compound, and was attached to the calorimeter chip. The calorimeter is suspended on thin phosphor-bronze wires and contains a resistive Joule heater on its backside and a Chromel - AuFe 0.07% thermocouple connected with its legs to the thermal bath. The heat capacity was measured by modulating the sample temperature periodically. A standard model of AC calorimetry^52^ relates amplitude and relative phase shift of the temperature modulation *T*_AC_ to the heat capacity (*C*_p_) of the sample and the thermal conductance of the wires on which the chip is suspended (*K*, determined in a separate calibration experiment): *T*_AC_ = *P*_0_/[*K* + iω *C*_p_] (*P*_0_ is the heating power ω the modulation frequency). If the frequency ω is chosen high enough (1–2 Hz for our calorimeter) the heat capacity dominates. In our experiment, the amplitude of the modulated heating power was kept constant. *T*_AC_ thus usually decreases with increasing temperature as a direct consequence of the increasing heat capacity of the sample. The amplitude of the heating power has been carefully adjusted so that the temperature modulation was always kept below 100 mK at the lowest temperature, and so that the signal-to-noise ratio was sufficiently large over the entire temperature range. Since the heat capacity is approximately inversely proportional to the modulation amplitude, the method naturally causes more noise in the data in the high temperature regime. All data presented in this manuscript have been taken upon slowly cooling the thermal bath from 8 K down to 1.4 K, while modulating the sample temperature with a typical modulation frequency of the order of 1 Hz. Note that the temperature modulation of the heat capacity chip causes a temperature offset to that the lowest sample temperature was 2 K. Since the data was taken upon very slow cooling with very small modulation amplitude, any overheating effects can be ruled out.

The small size of the superconducting specific heat anomaly (~1% of the total heat capacity of sample and calorimeter chip) required an extremely precise field-calibration of the thermocouple used in our calorimeter. This was done by comparing the specific heat of a silver calibration sample with and without applied magnetic field. To ensure significant precision, we only present data of the electronic specific heat derived from the difference in zero field data and 14 T data.

**Figure 9:**
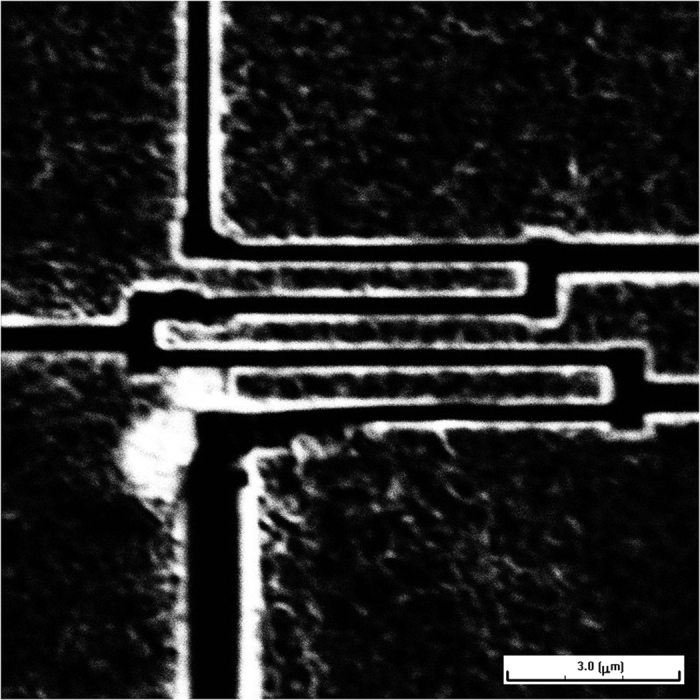
Electron microscopy image showing the separation of the metal layer deposited on a macroscopic polished grain formed by the network of Sn nanowires. The dark lines represents cuts from a focused ion beam used to separate various current and voltage terminals.

For the preparation of the devices for resistance measurements grains were extracted from the dried solution as described previously. A layer of 5 nm Ti and a layer of 70 nm Au was evaporated on a polished grain of the sample, which contains the loosely bound network of nanowire. The grain was fixed by epoxy on an insulating substrate. Four electrodes with a distance of 3.2 μm were separated with a focused ion beam technique. Figure 9 shows an image of the device configuration. The image shows the surface of the Au layer sputtered on top of the surface of the polished grain. The dark lines were cut through the metal by the focused ion beam thus exposing the nanowire network below. Note that on this micrometer scale no individual nanowires can be resolved, thus demonstrating the macroscopic length scale of this resistance measurement. The resistance measurement was carried out by sending an AC current of 20 nA amplitude and frequency of a few Hz generated by a Keithley Model 6221 AC and DC current source in combination with a Stanford Research RS830 digital lock-in amplifier. The experiments have been performed in a 15 T magnet cryostat with variable temperature inset that provides temperatures from 1.4 K–300 K.

## Additional Information

**How to cite this article**: Zhang, Y. *et al*. Dramatic enhancement of superconductivity in single-crystalline nanowire arrays of Sn. *Sci. Rep.* **6**, 32963; doi: 10.1038/srep32963 (2016).

## Supplementary Material

Supplementary Information
