# Supplementary material for: Dramatic enhancement of superconductivity in single-crystalline nanowire arrays of Sn
Source: Sci Rep. 2016 Sep 6;6:32963. doi: 10.1038/srep32963 (PMC5011740; doi:10.1038/srep32963)
Supplement: Supplementary Information [file srep32963-s1.pdf]

Dramatic enhancement of superconductivity in single-crystalline nanowire arrays of Sn

Ying Zhang[1], Chi Ho Wong[2,4], Junying Shen[2], Sin Ting Sze[2], Bing Zhang[2], Haijing Zhang[2], Yan Dong[1], Hui Xu[1], Zifeng Yan[1], Yingying Li[3], Xijun Hu[3] and Rolf Lortz[2]

[1] State Key Laboratory for Heavy Oil Processing, PetroChina Key Laboratory of Catalysis, China University of Petroleum, Qingdao 266580, China
[2] Department of Physics, Hong Kong University of Science and Technology, Clear Water Bay, Kowloon, Hong Kong
[3] Department of Chemical and Biomolecular Engineering, Hong Kong University of Science and Technology, Clear Water Bay, Kowloon, Hong Kong
[4] Institute of Physics and Technology, Ural Federal University, Russia

Supplementary Figures

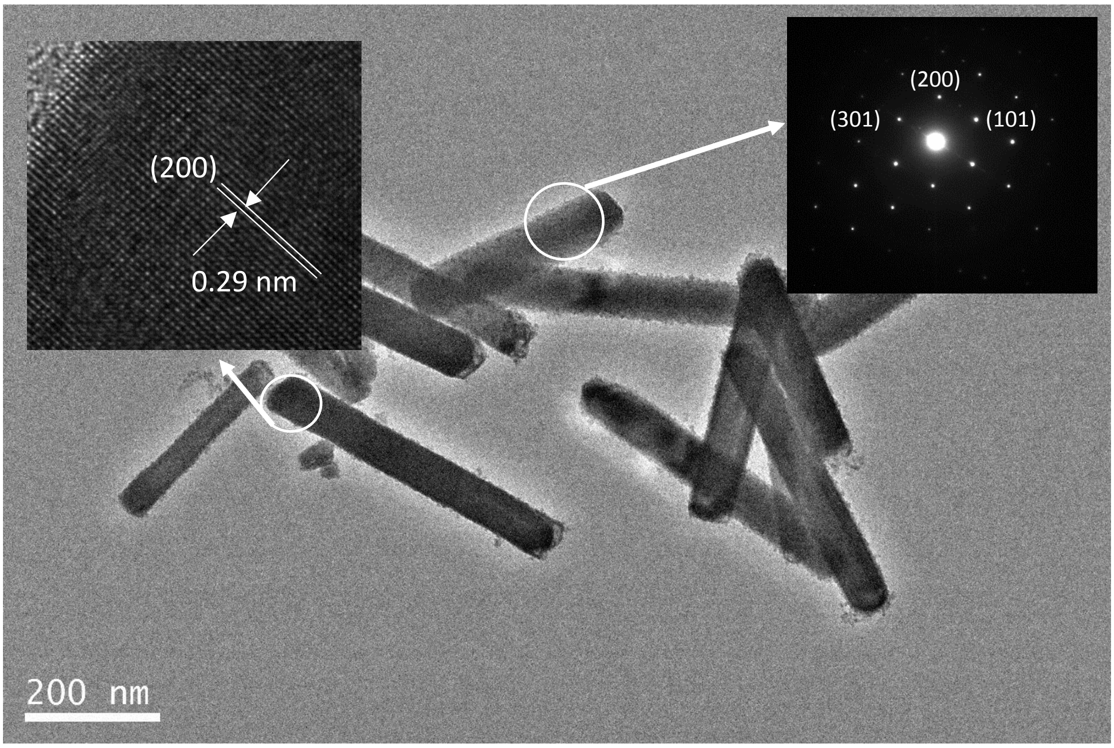

Supplementary Figure 1 | TEM image of a single Sn nanowire. The insets demonstrate the well-defined single crystalline structure of the nanowire in form of the corresponding SAED pattern and HRTEM images of the marked region
